# Polyol specificity of recombinant *Arabidopsis thaliana* sorbitol dehydrogenase studied by enzyme kinetics and in silico modeling

**DOI:** 10.3389/fpls.2015.00091

**Published:** 2015-02-23

**Authors:** M. Francisca Aguayo, Juan Carlos Cáceres, Matías Fuentealba, Rodrigo Muñoz, Claudia Stange, Ricardo Cabrera, Michael Handford

**Affiliations:** Departamento de Biología, Facultad de Ciencias, Universidad de ChileSantiago, Chile

**Keywords:** *Arabidopsis thaliana*, homology modeling, molecular dynamics simulation, pentavalent zinc, polyol

## Abstract

Polyols are enzymatically-produced plant compounds which can act as compatible solutes during periods of abiotic stress. Nicotinamide adenine dinucleotide^+^-dependent SORBITOL DEHYDROGENASE (SDH, E. C. 1.1.1.14) from *Arabidopsis thaliana* L. sorbitol dehydrogenase (AtSDH) is capable of oxidizing several polyols including sorbitol, ribitol, and xylitol. In the present study, enzymatic assays using recombinant AtSDH demonstrated a higher specificity constant for xylitol compared to sorbitol and ribitol, all of which are C2 (S) and C4 (R) polyols. Enzyme activity was reduced by preincubation with ethylenediaminetetraacetic acid, indicating a requirement for zinc ions. In humans, it has been proposed that sorbitol becomes part of a pentahedric coordination sphere of the catalytic zinc during the reaction mechanism. In order to determine the validity of this pentahedric coordination model in a plant SDH, homology modeling, and Molecular Dynamics simulations of AtSDH ternary complexes with the three polyols were performed using crystal structures of human and *Bemisia argentifolii* (Genn.) (Hemiptera: Aleyrodidae) SDHs as scaffolds. The results indicate that the differences in interaction with structural water molecules correlate very well with the observed enzymatic parameters, validate the proposed pentahedric coordination of the catalytic zinc ion in a plant SDH, and provide an explanation for why AtSDH shows a preference for polyols with a chirality of C2 (S) and C4 (R).

## INTRODUCTION

Nicotinamide adenine dinucleotide^+^-dependent SORBITOL DEHYDROGENASE (SDH, E. C. 1.1.1.14) is an enzyme required for the oxidation of inert sorbitol into metabolically-accessible fructose. Most SDH enzymes possess two zinc ions, one structural and the other catalytic. The mechanism proposed for the reaction of SDH with sorbitol requires that oxygen atoms of C1 and C2 are coordinated by a pentavalent catalytic zinc ion. The C2 hydroxyl group is thus in close proximity with C4 of the nicotinamide, leading to a chain of events which ultimately results in the reduction of NAD^+^ to NADH, and the formation of a C2 keto group in the fructose product (Pauly et al., 2003).

Sorbitol dehydrogenase has received substantial attention, because in several families such as Rosaceae and Plantaginaceae, sorbitol (also known as glucitol) is the principal photosynthate translocated through the phloem from source to sink organs (Zimmermann and Ziegler, 1975). The use of sorbitol is advantageous as more reducing power (NADH) is generated than if sucrose is transported and metabolized (Morandi et al., 2008). Additionally, sorbitol, and other metabolically-inert sugar alcohols are compatible solutes, levels of which increase during abiotic stress, including drought (Chen and Murata, 2002). In plants, SDH activity is high during the maturation of fruits of the Rosaceae family which import, but do not accumulate sorbitol, such as peaches (*Prunus persica*; Lo Bianco et al., 1999; Yamada et al., 2001), and Japanese pears (*Pyrus pyrifolia*; Oura et al., 2000) and cDNAs encoding SDHs from plum (*Prunus salicina*; Guo et al., 2012), and apple (*Malus* × *domestica*; Yamada et al., 1998; Park et al., 2002; Nosarzewski et al., 2004; Wang et al., 2009) have been cloned. Of these, extensive analyses of substrate specificity were performed using a purified pear form (Oura et al., 2000) and recombinant plum SDH (Guo et al., 2012). Both are multispecific, oxidizing sorbitol with highest relative activity (100%), whereas 5-carbon xylitol and ribitol were catalyzed less efficiently (76 and 14% for PpSDH; 62 and 15% for PsSDH, respectively).

Sorbitol dehydrogenase activity has also been identified in non-sorbitol translocating species including soybean (*Glycine max*, Fabaceae; Kuo et al., 1990) and maize (*Zea mays*, Poaceae; Doehlert, 1987). As in the case of SDHs characterized from the Rosaceae family, purified maize SDH, and a recombinant LeSDH from tomato (*Solanum lycopersicum*, Solanaceae) were also capable of oxidizing other polyols, albeit with lesser efficiency (Doehlert, 1987; Ohta et al., 2005). Recently, an SDH in the non-sorbitol translocating species *Arabidopsis thaliana* (Brassicaceae) has been identified and characterized (AtSDH, At5g51970; Nosarzewski et al., 2012; Aguayo et al., 2013). The use of mutants is enabling the physiological role of SDH to be elucidated in these species. For example, *atsdh-* mutants suffered reduced growth when supplemented with sorbitol (Aguayo et al., 2013). Additionally, under short day conditions, soil-grown mutants withstood drought stress better than wild-type plants, as shown by their enhanced relative water content and greater survival rates once rewatering had been resumed (Aguayo et al., 2013). These observations suggest that AtSDH is involved in metabolizing polyols which act as osmoprotectants and accumulate during drought stress. Although sucrose and raffinose are the main phloem translocated carbon sources in *Arabidopsis* (Haritatos et al., 2000), metabolic profiling studies have detected many different polyols such as glycerol, erythritol, xylitol, ribitol, mannitol, and sorbitol in this species (Fiehn et al., 2000; Kaplan et al., 2004; Rizhsky et al., 2004; Bais et al., 2010; Ebert et al., 2010). Of the polyols tested in enzyme assays, those oxidized preferentially by recombinant His-AtSDH were sorbitol (100%), ribitol (98%), and xylitol (80%; Aguayo et al., 2013). These three polyols all possess the same S and R configurations at C-2 (S) and C-4 (R), and it is the C2 hydroxyl group which is oxidized during their conversion to fructose, ribulose, and xylulose, respectively ^1^. Molecules with different configurations at these two C-atoms were oxidized by recombinant His-AtSDH at a lower rate [L-arabitol (C-2 (S), C-4 (S); 59%) and D-mannitol (C-2 (R), C-4 (R); 32%)] suggesting that this configuration is key for optimal catalytic activity (Oura et al., 2000; Aguayo et al., 2013). Interestingly, SDHs biochemically characterized from non-sorbitol translocating species, share the preference for sorbitol, whilst ribitol is oxidized at >60% of the efficiency of sorbitol (purified maize SDH, Doehlert, 1987; recombinant tomato LeSDH, Ohta et al., 2005). However, SDHs from Rosaceae species have a significantly lower ability to metabolize ribitol (<15% compared to sorbitol in apple (Negm and Loescher, 1979; Yamaguchi et al., 1994), pear (Oura et al., 2000) and recombinant plum (Guo et al., 2012).

In animals, SDH forms part of the polyol pathway, a means of converting glucose to fructose, via sorbitol (Jeffrey and Jornvall, 1983). Human SDH (HsSDH) oxidizes several polyols with similar relative efficiency, including sorbitol, xylitol, and ribitol (Maret and Auld, 1988). Several crystal structures of SDHs from different non-plant sources have been obtained. These include recombinant SDHs from silverleaf whitefly [*Bemisia argentifolii* (Genn.; Hemiptera: Aleyrodidae), PDB 1E3J, Banfield et al., 2001], human (PDB 1PL6, 1PL7, and 1PL8, Pauly et al., 2003) and *Rhodobacter sphaeroides* (PDB 1K2W, Philippsen et al., 2005). Of these structures, the one obtained from *R. sphaeroides* lacks zinc ions and substrates, BaSDH from whitefly contains both catalytic and structural zinc ions, but without substrates, and HsSDH (1PL6) is crystallized in the presence of NAD^+^, the catalytic zinc, and the inhibitor CP-166,572 showing interactions expected to resemble those achieved by sorbitol. Thus, a catalytic mechanism whereby the catalytic zinc changes from a tetrahedric coordination in the absence of substrates to a pentahedric geometry where hydroxyls 1 and 2 of sorbitol become part of the coordination sphere, was proposed (Pauly et al., 2003). More recently, an SDH from the liver of sheep (*Ovis aries*) has been crystallized (PDB 3QE3, Yennawar et al., 2011) in the presence of catalytic zinc. In this structure, an acetate molecule is observed close to the coordination sphere of the zinc atom, and a glycerol molecule is bound through hydrogen bonds with arginine, tyrosine, and glutamic acid residues of the binding pocket. In sheep SDH, it was proposed that only hydroxyl 1 of sorbitol contributes to the penta-coordination of zinc, establishing hydrogen bonds with the above-mentioned residues. Of note is that no crystal structure has yet been reported for the complex of a SDH with sorbitol in order to understand the role of zinc coordination, and the interactions with specific residues.

Homology modeling and Molecular Dynamic studies of the *Arabidopsis* enzyme could help to identify the key amino acid residues involved in substrate binding, and provide an explanation for the preference of the C-2 (S) and C-4 (R) configuration. Therefore, in order to understand the structural determinants of substrate specificity of AtSDH toward sorbitol, ribitol, and xylitol, the aim of this work was to correlate the kinetic performance of recombinant AtSDH toward these three substrates, with the dynamic behavior of their respective interactions observed in Molecular Dynamics simulations.

## MATERIALS AND METHODS

### EXPRESSION AND PURIFICATION OF RECOMBINANT HIS-AtSDH AND AtSDH

*Arabidopsis thaliana* sorbitol dehydrogenase fused at its N-terminus to a 6xHis tag (His-AtSDH; Aguayo et al., 2013), was expressed *in vitro* from the pEXP5-NT/TOPO vector using the Expressway Cell-Free expression system (Invitrogen) according to the manufacturer’s instructions with minor modifications (1.5 μg of plasmid DNA per reaction; expression at 30^∘^C for 6 h). For purification, 10 parallel *in vitro* reactions (250 μl each) were resuspended in binding buffer (50 mM Tris-HCl pH 8.2, 500 mM NaCl, 10 mM imidazole, 10% glycerol) and loaded onto a His-Spin Protein Miniprep (Zymo-Research) and the column washed with four volumes of binding buffer containing 50 mM imidazole. Bound proteins were eluted with binding buffer containing 250 mM imidazole as described previously (Aguayo et al., 2013). The N-terminal His tag was then removed by adding 1 mg TEV protease to 5 mg His-AtSDH and incubating at 25^∘^C for 2 h in three volumes of 50 mM Tris-HCl pH 8.2, 1 mM T-CEP and 10% glycerol. The imidazole was removed from the TEV protease buffer at the end of the incubation after two cycles of dilution in four volumes of 50 mM Tris-HCl pH 8.2, 500 mM NaCl and 10% glycerol, and concentration through a Centricon column (10 kDa Millipore, 15 min, 4500 × *g*). The concentrated protein mix (40–80 ng/μl in buffer without imidazole) was then loaded onto a His-Spin Protein Miniprep and the flow-through fraction (containing recombinant AtSDH) collected for further experiments. In multiple experiments, the recovery of recombinant AtSDH (1 mg) was approximately 20% of the recombinant His-AtSDH (5 mg) originally synthesized. The recombinant proteins were separated by SDS-PAGE, visualized by Coomassie staining and detected by immunoblot analysis using monoclonal anti-His (Sigma; to detect His-AtSDH) antisera and anti-mouse alkaline phosphatase-conjugated secondary (Sigma) antisera.

### ENZYMATIC ANALYSIS OF RECOMBINANT AtSDH

Dehydrogenase activity was determined spectrophotometrically by measuring the rate of change in absorbance at 340 nm for NAD^+^ reduction at 25^∘^C, using a Unicam spectrophotometer (model UV2). Reactions were initiated by adding purified recombinant His-AtSDH or AtSDH (1.2–1.5 μg) to a standard reaction mixture containing 100 mM Tris-HCl pH 9, 20 mM polyol and 1.36 mM NAD^+^ (as determined by enzymatic titration). In separate experiments, sorbitol, ribitol, xylitol, and NAD^+^ concentrations were varied in order to determine the respective kinetic parameters, using enzyme collected from at least three independent *in vitro* expression reactions and purifications. The initial velocity (*v*) was determined at the different substrate concentrations, [S]. In the case of sorbitol and ribitol, the *K*_m_ was calculated by fitting to the Michaelis–Menten hyperbolic function: *v* = V_max_ [S]/(*K*_m_ + [S]), and *k*cat was determined using the following equation: *k*cat = V_max_/[E]_tot_ , where [E]_tot_ refers to the total enzyme amount. In the case of xylitol, the following equation was used, incorporating substrate inhibition: *v* = V_max_ [S]/(*K*_m_+[S]([S]^2^)/*K*_i_); Cornish-Bowden, 2012). All data were fitted using SigmaPlot (Systat Software, San Jose, CA, USA), which uses non linear regression by an iterative least squares algorithm for parameter estimation.

### MOLECULAR DOCKING

The PDB file of the HsSDH structure (PDB 1PL6, Pauly et al., 2003) was modified by changing selenomethionine residues to methionine, and selecting for the highest occupancy of those methionine residues with multiple conformations. Additional preparation of the structure was performed by using AutoDock Tools (Morris et al., 2009). A +2 charge was assigned to the structural and catalytic zinc atoms, and sorbitol, ribitol, and xylitol were positioned for flexible docking using the ideal conformation for these ligands from Ligand depo (Feng et al., 2004). The chirality of the carbon atoms was confirmed according to the ChEBI database (Hastings et al., 2013). The docking calculations were performed using Autodock Vina, with 250 as the exhaustiveness parameter. The docking area was defined by a box (17 Å × 15 Å × 15 Å) centered on the location of the CP-166,572 inhibitor molecule present in the HsSDH structure (Pauly et al., 2003). Twenty different conformations were generated for each polyol which were ranked according to their binding energy, in agreement with the expected coordination of pentavalent catalytic zinc and proximity between C2 of the polyol and C4 of the nicotinamide moiety of NAD^+^ (where the hydride is transferred) in order to select the best template for further modeling.

### *IN SILICO* MODELING

Two types of model were generated; (i) NAD^+^-bound AtSDH (without polyols) with two zinc atoms, one associated with the catalytic site and the other structural; (ii) NAD^+^-bound AtSDH with two zinc atoms, one associated with the catalytic site, and the other structural, and a polyol substrate (sorbitol, xylitol, or ribitol).

For the first model, the structure of HsSDH was used as template for the active site containing NAD^+^ and the tetra-coordinated catalytic zinc (PDB 1PL8; Pauly et al., 2003). The second template was the structure of BaSDH (PDB 1E3J; Banfield et al., 2001) to contribute the structural zinc. Therefore, in the case of the second model type, the polyol-bound form generated by docking of HsSDH (see Molecular Docking) was the template which contributed the substrates and the catalytic zinc, whilst the structure of BaSDH contributed the structural zinc.

The alignment of the amino acid sequences of AtSDH, HsSDH, and BaSDH was performed using ClustalX 2.1 (Larkin et al., 2007), and the result served as the input for the generation of three-dimensional models using Modeler 9.11 (Sali and Blundell, 1993; Eswar et al., 2008). The resulting alignment showed that the first 18 amino acids at the N-terminus of AtSDH have no equivalent in the other templates. Ten models were generated for each complex, employing methods of conjugate gradients, and molecular simulation with simulated annealing, performed by Modeler. The quality of the best model was assessed by the determination of its energy (ProsaII; Sippl, 1993) and local sequence-structure correlation (Verify3D; Eisenberg et al., 1997). In the case of NAD^+^-bound AtSDH, the structure of the 18 amino acids at the N-terminus was predicted by Jpred3 (Cole et al., 2008) and then subjected to *ab initio* modeling using the GalaxyWeb server (Ko et al., 2012). The GalaxyLoop procedure (Park et al., 2011) was employed to refine the region between amino acids 1 and 18, using the PS1tbm scoring method. Five models were obtained and the best one was chosen according to the same evaluation criteria mentioned above. In the case of the enzyme-substrate complexes, the first 18 amino acids were not included in the final models (see Modeling NAD^+^-Bound AtSDH).

### MOLECULAR DYNAMICS SIMULATION

Ten nanosecond trajectories were simulated for the generated models of NAD^+^- bound AtSDH, and NAD^+^- bound AtSDH in complex with sorbitol, ribitol, or xylitol, using NAMD 2.8 (Phillips et al., 2005) and the force field AMBERff99SB (Hornak et al., 2006). Systems were prepared with Ambertools 1.5 (Case et al., 2010). In the case of the polyols, the parameters and topologies were generated by homology using Antechamber (Wang et al., 2006), whereas previously-described parameters and topologies were used in the case of NAD^+^ (Ryde, 1995). Each system was simulated in a box of TIP3P waters with a pad of 13 Å in all directions and the overall charge of the system was neutralized using three Na^+^ ions. Integration steps of 1 fs were used, and non-bound interactions were considered within a radius of 9 Å, with a switching function over 11 Å. For long range interactions, the Particle–Mesh Ewald model was employed (Darden et al., 1993). For each system, 100,000 steps of energy minimization were applied, followed by a gradual temperature increase to 300 K.

Unlike the tetrahedric coordination of zinc, to the best of our knowledge the parameters needed to simulate the pentahedric coordination of zinc are not defined in the force field used or elsewhere. Therefore, harmonic restrictions on the distances and angles between the atom ligands around the catalytic (Cys36, His61, HO-, O1, and O2 of the polyols) and structural (Cys91, Cys94, Cys97, Cys105) zinc atoms were applied, according to the regular distances and coordination angles observed in crystallographic structures (Alberts et al., 1998). In addition, the Glu62 residue was set to its protonated form in order to prevent its tendency to interact with the zinc atom, which in turn disturbs the coordination geometry. In the case of NAD^+^-bound AtSDH, both the catalytic and structural zinc atoms were modeled with tetrahedric coordination. For the simulations, harmonic distance and angle restrictions were applied on the four molecules involved in their coordination (Cys36, His61, HO-, and Glu62 for the catalytic zinc; Cys91, Cys94, Cys97, and Cys105 for the structural zinc).

VMD 1.9 (Humphrey et al., 1996) was used to analyze trajectories. Hydrogen bonds were quantified using a cut-off distance of 4 Å, with an Acceptor-Hydrogen-Donor angle greater than 120^∘^. The radial pair distribution function was calculated between the polyols and the oxygen atoms of the water molecules at 3 Å from the protein. The Stamp tool from the MultiSeq package (Roberts et al., 2006) was used to perform structural superpositions between the docking complexes of HsSDH and the models of AtSDH complexes after 100,000 minimization steps.

## RESULTS AND DISCUSSION

### EXPRESSION AND PURIFICATION OF RECOMBINANT HIS-AtSDH and AtSDH

**FIGURE 1:**
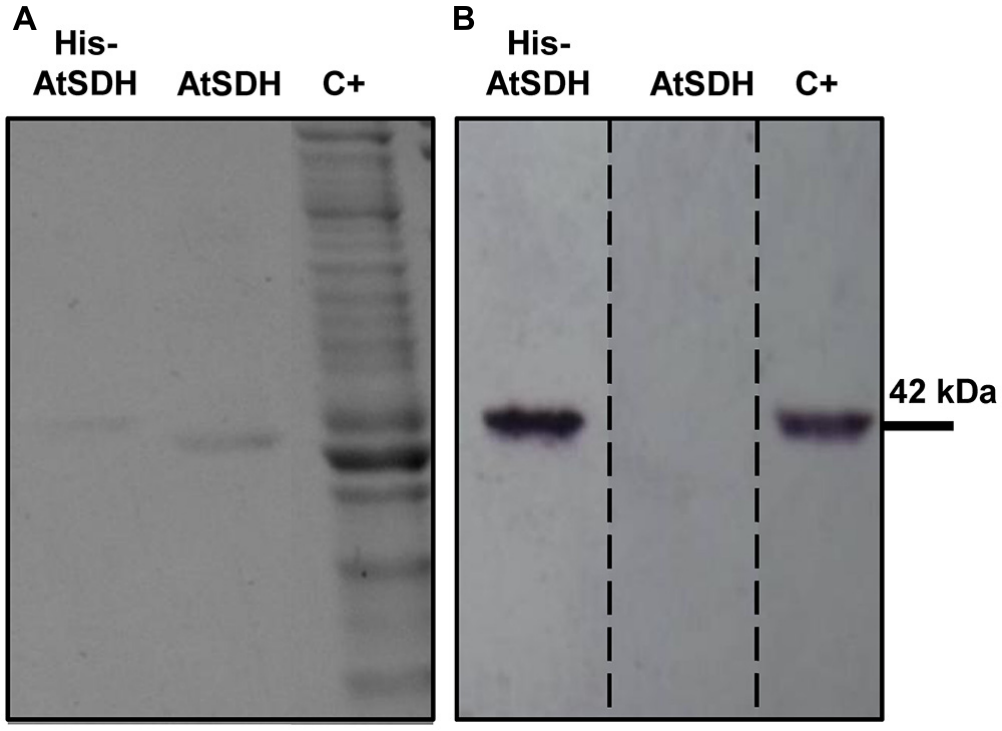
**Expression and purification of recombinant His-AtSDH and AtSDH. (A)** Coomassie stained gels containing purified recombinant AtSDH, before (lanes His-AtSDH) and after (lanes AtSDH) TEV-mediated cleavage of the His-tag; note the difference in migration of recombinant His-AtSDH vs. AtSDH. C+; whole reaction products from the *in vitro* expression of recombinant His-AtSDH. **(B)** Immunoblot analysis of the samples in **(A)**, using an anti-His antisera. The dashed lines denote that this image is a reconstruction of the original immunoblot, which is shown in Supplementary Figure S1.

Previously, we showed that recombinant His-AtSDH is capable of oxidizing a variety of linear polyols, exhibiting greatest specific activities with sorbitol, ribitol, and xylitol (Aguayo et al., 2013). In the present study, we chose to continue working with His-AtSDH expressed *in vitro*, as recombinant tagged versions expressed in *Escherichia coli* or *Saccharomyces cerevisiae* formed inclusion bodies with no enzymatic activity post-solubilization or lost activity during the purification process, respectively (unpublished results). In order to determine the kinetic parameters of the recombinant form of the enzyme with these three substrates, recombinant His-AtSDH and AtSDH were expressed and purified, as described in Materials and Methods. The increased migration in SDS-PAGE, coupled with the lack of cross reactivity with the anti-His antisera in immunoblot assays indicated that the His-tag had been completely removed from AtSDH by TEV protease treatment (**Figures 1A,B**, Supplementary Figure S1). On using sorbitol as substrate at a saturating NAD^+^ concentration (1.36 mM), it was noted that the *K*_m_ remained relatively unchanged by the excision of the His-tag [His-AtSDH: 1.20 ± 0.16 mM, three replicates (Aguayo et al., 2013); AtSDH: 0.96 ± 0.07 mM, three replicates]. Such a minor effect on the affinity for sorbitol was also observed on removal of a Maltose Binding Protein from the N-terminus of purified recombinant SDH from plum (Guo et al., 2012). However, the turnover number of the *Arabidopsis* enzyme increased more than sixfold when the His-tag was removed [His-AtSDH: 0.33 ± 0.01 s^-1^, three replicates (Aguayo et al., 2013); AtSDH: 2.13 ± 0.03 s^-1^, three replicates]. The difference between these two versions is discussed below, and we proceeded with the kinetic characterization using recombinant AtSDH, as this form is a truer representation of the enzymatic parameters observed for the enzyme *in planta*.

### POLYOL SPECIFICITY OF RECOMBINANT AtSDH

**FIGURE 2:**
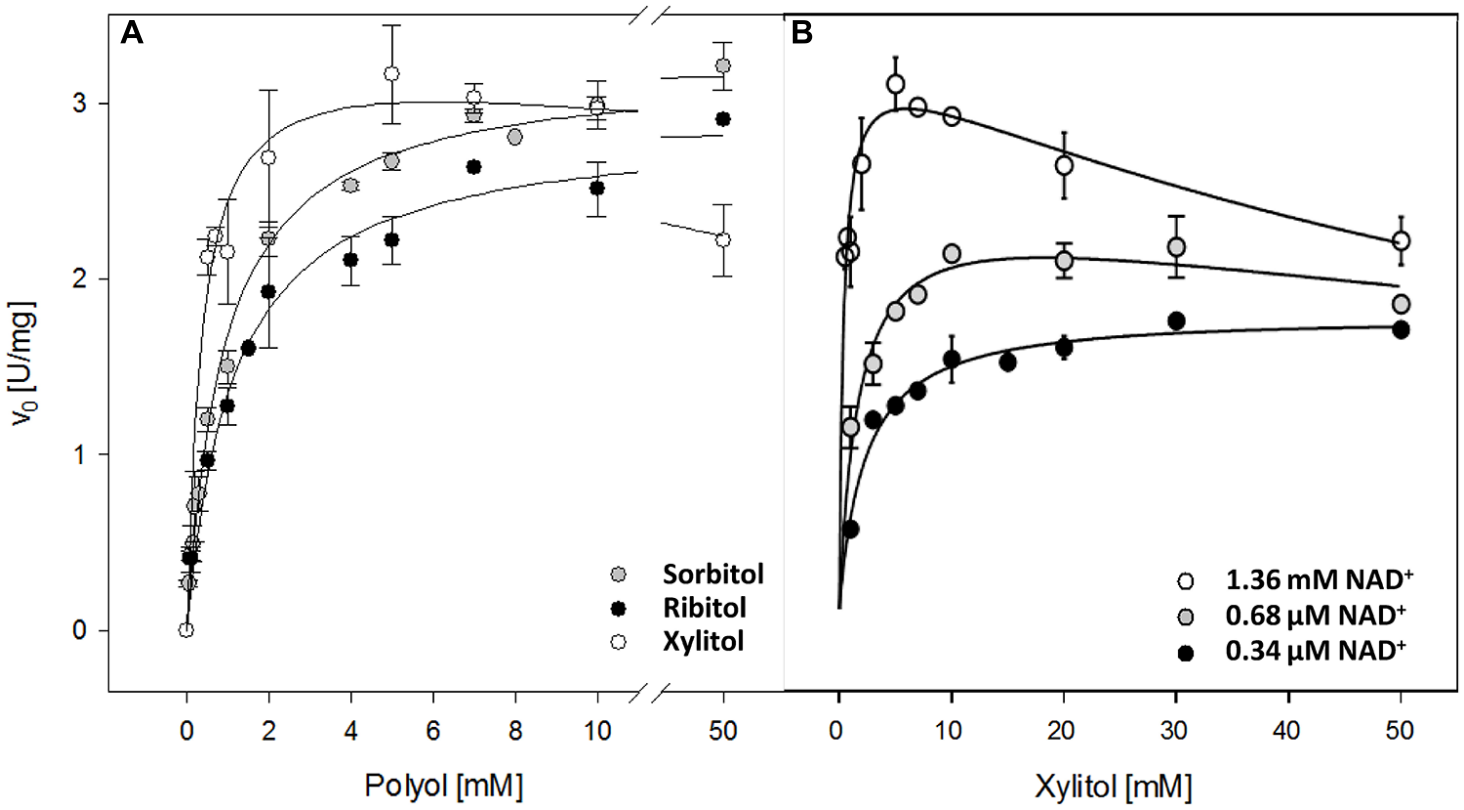
**Kinetic properties of recombinant AtSDH. (A)** Specific activities were obtained with sorbitol, ribitol, and xylitol as the variable substrate, whilst the co-substrate concentration was held saturating at 1.36 mM NAD^+^. The continuous lines represent the fit to a hyperbolic function. The values are the means ± SD of at least three independent determinations. **(B)** Specific activities were obtained with xylitol as the variable substrate, whilst the co-substrate concentration was held at 34 μM, 68 μM, or 1.36 mM NAD^+^. The continuous lines represent the fit to a substrate inhibition model (not shown). The values are the means ± SE of three independent determinations.

The *K*_m_ of recombinant AtSDH with sorbitol and ribitol as substrates were similar (**Figure 2A**; **Table 1**), as were the turnover numbers for all three substrates (**Table 1**) at 1.36 mM NAD^+^. These results confirm that AtSDH also acts as a ribitol dehydrogenase and a xylitol dehydrogenase, and are consistent with the observation that in long day conditions, *atsdh-*mutants possess elevated levels of sorbitol and ribitol (Nosarzewski et al., 2012; Aguayo et al., 2013). The apparent affinity of recombinant AtSDH for sorbitol (*K*_m_ 0.96 mM) was very similar to that determined for HsSDH (*K*_m_ 0.62 mM; Maret and Auld, 1988), lower than that observed in other non-Rosaceae species such as recombinant tomato SDH (*K*_m_ 2.39 mM; Ohta et al., 2005) and purified maize SDH (*K*_m_ 8.45 mM; Doehlert, 1987), and several orders of magnitude lower than that of recombinant or partially purified SDHs studied in Rosaceae species such as plum (*K*_m_ 111.8 mM; Guo et al., 2012), Japanese pear (*K*_m_ 96.4 mM; Oura et al., 2000), apple (*K*_m_ 86 mM; Negm and Loescher, 1979) and peach (*S*_0.5_ 43 mM; Hartman et al., 2014). Of the three polyols evaluated, recombinant AtSDH exhibits the highest specificity constant with xylitol, yet the specific activity with this substrate was lower than that of sorbitol and ribitol when recombinant His-AtSDH was used (Aguayo et al., 2013). We believe that this can be attributed to the fact that the latter assays were performed at 2 mM NAD^+^ and 50 mM xylitol, concentrations which produce substrate inhibition, as shown in **Figure 2B**. Nevertheless, the *K*_m_ of recombinant AtSDH with xylitol (0.27 mM) is very similar to that of HsSDH (0.22 mM; Maret and Auld, 1988) and substantially lower than with the other substrates, as also found in purified apple SDH (37 mM; Negm and Loescher, 1979), translating into a higher specificity constant with this 5-carbon molecule than with either sorbitol or ribitol (**Figure 2A**; **Table 1**). At xylitol concentrations greater than 5 mM in the presence of 1.36 mM NAD^+^, the specific activity was significantly inhibited, a phenomenon not observed with the other two polyol substrates. However, when substrate inhibition is considered in the fitting of initial velocity data (not shown) the *K*_i_ was far beyond physiological concentrations of NAD^+^ (more than 100 mM). This property of recombinant AtSDH with xylitol was reduced at a lower NAD^+^ concentration, and absent at 34 μM NAD^+^ (**Figure 2B**). The phenomenon of substrate inhibition in oligomeric enzymes could originate from the negative interaction between the active sites or the presence of allosteric sites (Johnson and Reinhart, 1992; Cabrera et al., 2008). In order to determine the quaternary structure of the plant enzyme, repeated attempts were made to obtain sufficiently-concentrated recombinant AtSDH for performing gel filtration chromatography. However, these efforts were unsuccessful due to the propensity of the purified enzyme to precipitate when concentrated under the experimental conditions employed. Therefore, although it is not known if AtSDH functions as an oligomer, other SDHs have a tetrameric quaternary structure (e.g., HsSDH, Pauly et al., 2003; sheep SDH, Yennawar et al., 2011). If AtSDH does function in a quaternary state, then the binding of NAD^+^/xylitol at one site could negatively affect the affinity for these substrates at others due to long range coupling through oligomeric packing, thus leading to the inhibition of xylitol oxidation at higher NAD^+^ concentrations. However we consider that this phenomenon is more likely to be a side effect of the elevated NAD^+^ concentrations involved, which are not physiologically relevant *in planta*.

**Table 1:** Kinetic properties of recombinant AtSDH.

Substrate	*K*_m_ (mM)	*k*cat (s^-1^)	*k*cat/*k*_m_ (mM^-1^ s^-1^)
Sorbitol	0.96 ± 0.07	2.13 ± 0.03	2.23 ± 0.17
Ribitol	1.05 ± 0.10	1.83 ± 0.03	1.74 ± 0.17
Xylitol	0.27 ± 0.04	2.12 ± 0.06	7.74 ± 1.01

The *K*_m_, *k*cat and specificity constant (*k*cat/*K*_m_) of recombinant AtSDH with sorbitol, ribitol, and xylitol were determined at a constant co-substrate concentration (1.36 mM NAD^+^). Values are means ± SE of at least three independent determinations.

Sorbitol dehydrogenase enzymes from most species harbor two zinc atoms, one structural and the other at the active site. In line with this finding, in plants it has been shown that the activity of a recombinant peach SDH increases dramatically if the bacterial cells are grown in the presence of zinc chloride (Hartman et al., 2014). Therefore, in order to determine whether AtSDH also possesses coordinated zinc molecules, the enzyme was pre-incubated with varying concentrations of the divalent ion chelator, EDTA, and then evaluated for activity. The specific activity of AtSDH with sorbitol (20 mM) and NAD^+^ (0.34 mM) fell by 48% after preincubation with 1 mM EDTA for just 60 min, compared to the enzyme pre-incubated in the absence of EDTA, strongly indicating that zinc does indeed play a key role in the enzymatic activity of the plant enzyme.

### MODELING NAD^+^-BOUND AtSDH

The full 364-amino acid sequence of AtSDH was used to perform a BLAST search (Altschul et al., 1990) of the PDB ^2^ in order to obtain suitable templates. The first five hits obtained correspond to SDHs, as shown in Supplementary Table S1. In order to generate a structure of NAD^+^-bound AtSDH, two templates were chosen, both of which share >46% amino acid identity with the plant enzyme. Specifically, we used the structure of BaSDH (PDB 1E3J; Banfield et al., 2001), because it is the only SDH template present in PDB that contains the structural zinc atom. We also used the HsSDH structure (PDB 1PL8; Pauly et al., 2003), due to the presence of NAD^+^ and catalytic zinc. Sequence alignments of plant SDHs (Nosarzewski et al., 2012; Aguayo et al., 2013; Hartman et al., 2014) show that the AtSDH protein sequence possesses the four conserved Cys residues involved in the binding of the structural zinc.

**FIGURE 3:**
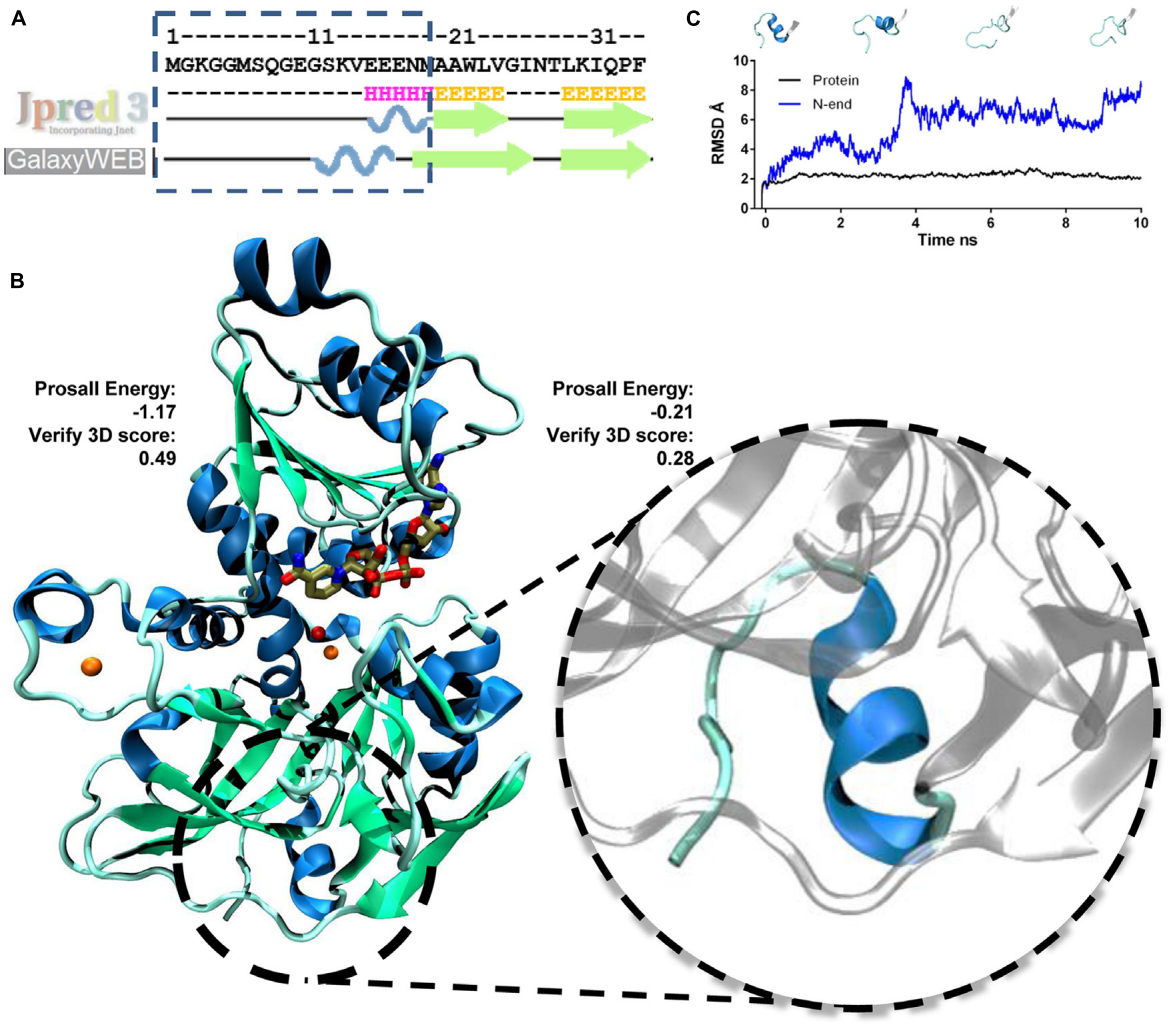
**Modeling the structure of NAD^+^-bound AtSDH. (A)** Secondary structure prediction of the N-terminus of AtSDH. The prediction was performed using Jpred3 and the secondary structure observed after modeling *ab initio* by loop refinement (GalaxyWeb). **(B)** 3D representation of a monomer of NAD^+^-bound AtSDH (blue ribbons) obtained by homology modeling followed by *ab initio* modeling of the 18 amino acid residues at the N-terminus (blue ribbon enlarged within the dashed circle). The results of the ProsaII energy and Verify3D scores for the N-terminus and the rest of the protein are shown. The structural and catalytic zinc atoms are colored orange, NAD^+^ is colored brown and a water molecule coordinated by the catalytic zinc atom is shown in red. **(C)** RMSD graph of the N-terminus (blue line) and of NAD^+^-bound AtSDH without the N-terminus (black line) during a 10 ns simulation. Snapshots of modeled structures of the N-terminus are shown at different time points.

The first 18 amino acids of the N-terminal of AtSDH do not align with the sequences of either template. In an updated phylogenetic analysis of more than 40 known and putative SDHs from mono- and dicotyledonous species (Hartman et al., 2014), all except that from *Triticum urartu* possess an N-terminal extension compared to these non-plant templates. Therefore, in order to determine whether this region could be structured in plant SDHs, homology modeling and *ab initio* loop refinement of the N-terminal 18 amino acids of AtSDH were performed. As a first step, the evaluation of the resulting model via ProsaII resulted in a Z-score with a value expected for a protein of 364 amino acid residues (-9.03), and Verify3D analysis gave a score of 93.7%, again with a good local correspondence between sequence and structure. In the second stage, the structure of these 18 amino acids was refined. On performing a prediction of the secondary structure derived from the AtSDH sequence, a high probability for the formation of an α-helix at the N-terminus was determined (**Figure 3A**). Five *ab initio* models were obtained, of which the best possessed a *Z*-score of -8.45, and 96.16% of the residues had a good Verify3D score. However, the residues at the N-terminus have an average ProsaII value of -0.21, compared to -1.17 for the rest of AtSDH, indicating that the structure formed by the 18 amino acids at the N-terminus is less favorable. Nevertheless, the Verify3D score of these residues is less than that of the entire protein (0.28 vs. 0.49, respectively), indicating that the correspondence between sequence and structure is not good in this region. As shown in **Figure 3B**, the final structure predicted from the primary sequence is an α-helix.

The stability of the model generated was analyzed by a Molecular Dynamics simulation for 10 ns. The full 364-amino acid sequence of AtSDH is highly stable during the trajectory, with a RMSD of 2.5 ± 0.3. However, the *ab initio* modeled N-terminus is less stable; the RMSD of the first 18 amino acids is 5.6 ± 1.6, whereas that of the remainder of the protein (346 amino acids) is just 2.21 ± 0.19 (**Figure 3C**). The N-terminus tends to unfold during the simulations; after 1 ns, the α-helix presents one turn less, and after 4 ns, the helix is completely unwound and does not form again during the 10 ns trajectory (**Figure 3C**). However, the immediate downstream secondary structure, a β-strand, maintains its structure and position throughout the simulation.

Given the predicted unpacking and loosening of the N-terminus of AtSDH, this zone of the polypeptide chain may indeed be less-structured, or maybe the model does not capture effectively the native configuration of the N-terminus. In addition, the N-terminal region is located approximately 28 Å from the active site, meaning it is unlikely that it participates directly in substrate binding and/or catalysis. Considering that other SDHs function as tetramers (HsSDH, Pauly et al., 2003; sheep SDH, Yennawar et al., 2011; pear SDH, Oura et al., 2000; plum SDH, Guo et al., 2012; peach SDH, Hartman et al., 2014), the predicted AtSDH structure was superimposed onto the tetrameric structure of HsSDH (Pauly et al., 2003). The overlay shows that the N-terminus is unlikely to participate in interface interactions, nor is it close to the active sites of the neighboring subunits.

Considering the observed effects of the presence of the His-tag on enzymatic activity (see Expression and Purification of Recombinant His-AtSDH and AtSDH), it appears that this tag is a cause of unfavorable consequences. For example, it has been previously reported that the proximity of the His-tag to cysteine residues in recombinant corticotropin-releasing factor receptor affected the formation of disulfide bridges (Klose et al., 2004). However, around 90% of the crystallized proteins whose structures are deposited in PDB correspond to recombinant proteins (including both HsSDH and BaSDH), and of these recombinant proteins, 60% have been purified by means of a His-tag (Carson et al., 2007). Therefore, we consider that removal of this tag produces a more faithful model of the native protein, and for these reasons, the first 18 amino acids of AtSDH were not included during the Molecular Dynamics simulations in the presence of the polyol substrates.

### MODELING OF INTERACTIONS OF AtSDH TERNARY COMPLEXES WITH DIFFERENT POLYOLS

**FIGURE 4:**
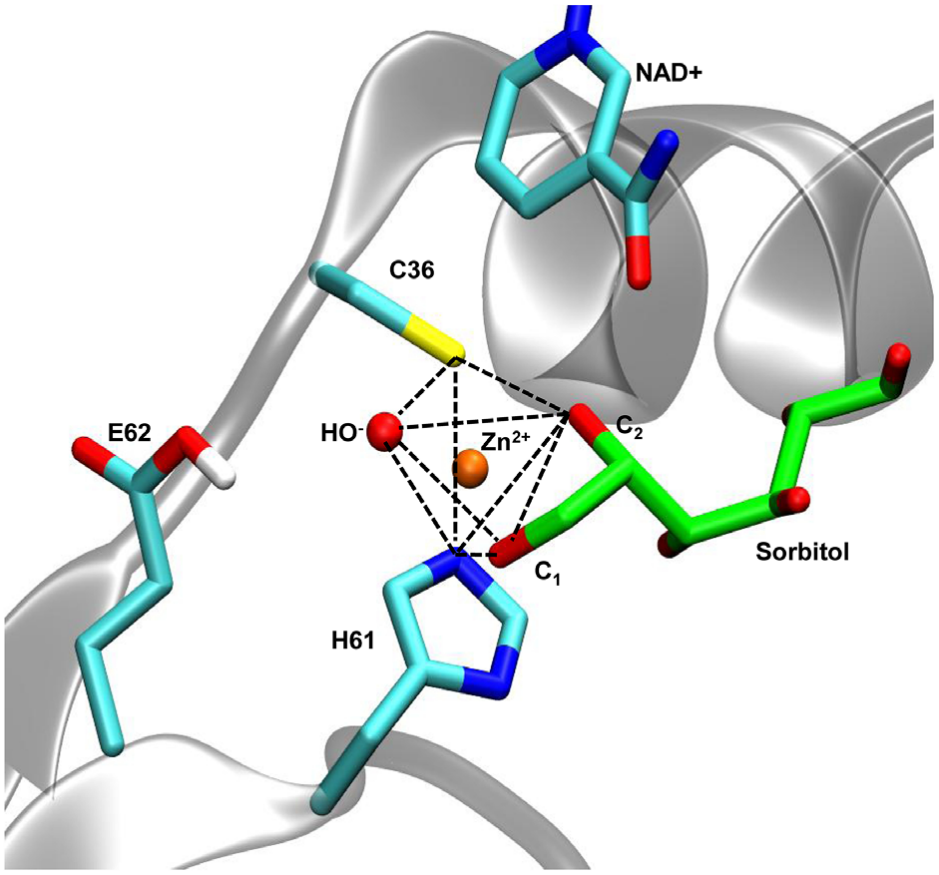
**Coordination geometry of the catalytic zinc of AtSDH in complex with sorbitol.** In AtSDH (gray cartoon), Sγ of Cys36, N𝜀 of His61, and an oxygen atom of a water molecule form part of the trigonal bipyramidal coordination of the catalytic zinc (dashed black lines); the O1 and O2 atoms of sorbitol complete the coordination sphere. The sidechain of Glu62 was simulated in a protonated state to avoid interference with the penta coordination (see Modeling of Interactions of AtSDH Ternary Complexes with Different Polyols). The nicotinamide moiety of NAD+ is shown in close proximity to the polyol.

Since we adhere to the proposal by Pauly et al. (2003) about the role of sorbitol hydroxyls 1 and 2 in the coordination of catalytic zinc, and given that recombinant AtSDH has similar kinetic properties compared to HsSDH (see Polyol Specificity of Recombinant AtSDH), we chose 1PL6, the structure of HsSDH, as the scaffold to prepare the coordinates of the template polyol complexes. Unlike 1PL8, in 1PL6 the inhibitor molecule CP-166,572 is observed participating in the trigonal bipyramidal coordination of the catalytic zinc (Supplementary Table S1). The inhibition exercised by CP-166,572 is competitive and uncompetitive with respect to fructose and sorbitol, respectively (Pauly et al., 2003). Nevertheless, the catalytic mechanism proposed for HsSDH indicates that all three molecules occupy the same physical space, and notably the same two hydroxyl groups (1 and 2) of fructose and sorbitol coordinate the catalytic zinc (Pauly et al., 2003). Additionally, the NAD^+^ molecule in this structure was kept as the template for modeling this cofactor in AtSDH. We obtained the structures of HsSDH in complex with sorbitol, ribitol, or xylitol by molecular docking, using the site occupied by the inhibitor in 1PL6 as the docking space. Interestingly, most of the resulting conformations and those with the lowest binding energies present hydroxyls 1 and 2 of the polyols orientated toward the catalytic zinc. **Figure 4** shows the best docking solution (see Materials and Methods) that was used as one of the templates for modeling the AtSDH complexes.

The evaluation of the resulting AtSDH models showed acceptable *Z*-scores which were within the values expected for the length of AtSDH (-9.11, -9.15, -9.12 for sorbitol, ribitol, and xylitol complexes, respectively; Sippl, 1993 ^3^). The analysis by Verify 3D showed that more than 85, 91, and 88% of the residues in sorbitol, ribitol, and xylitol complexes, respectively, present scores indicating good local correspondence between sequence and tertiary structure (Supplementary Figure S2).

A protocol of energy minimization was applied to all the models to improve the packing of sidechains. The coordination geometry of the catalytic zinc involved five atom ligands: the sulfur atom of Cys36, the hydroxyls of C1 and C2 in the polyol, the nitrogen N𝜀 of His61 and a structural water (**Figure 4**). In the following stage of molecular simulation analysis, an important consideration was taken for the protonation state of Glu62. Different preliminary simulations showed that the negatively-charged carboxylate tends to interfere with the penta-coordinated ligands due to the coulombic attraction with the positively-charged zinc. The systems behaved with greater stability when Glu62 was in a protonated state. Additionally, to maintain the consistency with the reaction mechanism proposed for HsSDH, with water functioning as a general base (Pauly et al., 2003), we deprotonated the structural water in the coordination sphere. Thus, we maintained these criteria during all the subsequent minimization and molecular simulation steps.

**FIGURE 5:**
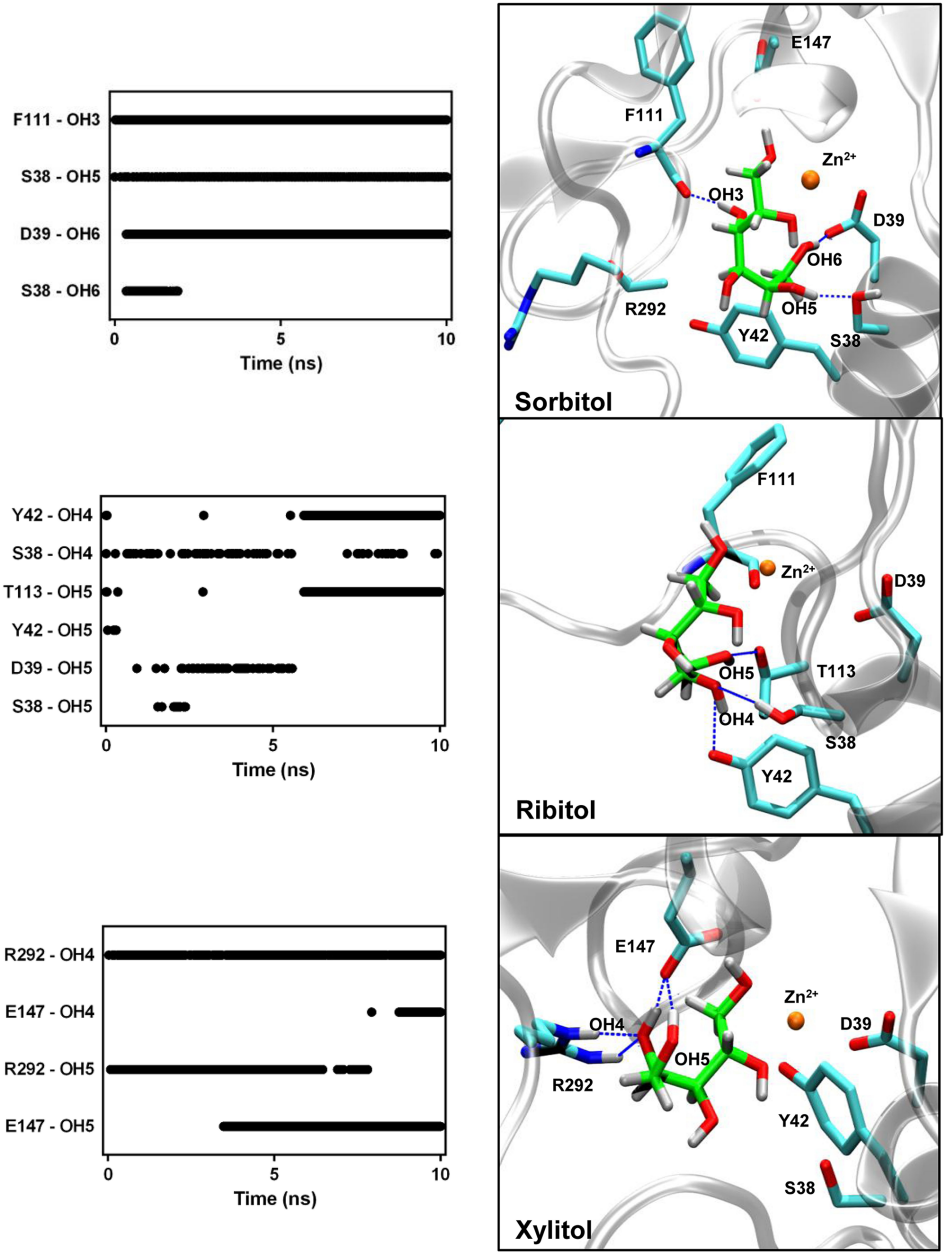
**Hydrogen bond interactions between the AtSDH models and the polyols.** The hydrogen bonds formed between AtSDH and sorbitol, ribitol, or xylitol, were quantified every 25 ps during the trajectories (left hand panels). The protein residues of the binding pocket identified in each case are represented in the right hand panels, in which dashed blue lines mark those hydrogen bonds that were maintained for at least 1 ns. The interactions with the arginine guanidinium group (R292) exhibit bidentation, hence contributing with two effective hydrogen bonds. The colors of the atoms are the same as described in **Figure 3**.

We first analyzed the time course of direct hydrogen bonds between the polyols and the protein residues during 10 ns simulation trajectories. The greatest numbers of interactions were observed for xylitol with an average of 5.4 ± 1.3 bonds and sorbitol with an average of 4.9 ± 0.9 bonds. In contrast, ribitol showed an average of 2.4 ± 0.9 hydrogen bonds with AtSDH. **Figure 5** shows that the hydrogen bonds formed with the polyols involve in the case of sorbitol, the interaction between hydroxyls 3, 5, and 6 with residues Phe111, Ser38, and Asp39, respectively; in the case of xylitol, hydroxyls 4, and 5 interacting with Arg292 and Glu147, respectively; and in the case of ribitol, hydrogen bonds are formed rather transiently between hydroxyl groups 4 and 5 and residues Thr113, Tyr42, Asp39, and Ser38. Considering that the *K*_m_ of recombinant AtSDH is substantially lower than that of other plant SDHs (see Expression and Purification of Recombinant His-AtSDH and AtSDH), evaluating *in silico* the conservation and orientation of these residues in characterized SDHs of plant origin, and subsequent site-directed mutagenesis studies will be informative in determining their true relevance in the substrate specificity and performance of these enzymes.

**FIGURE 6:**
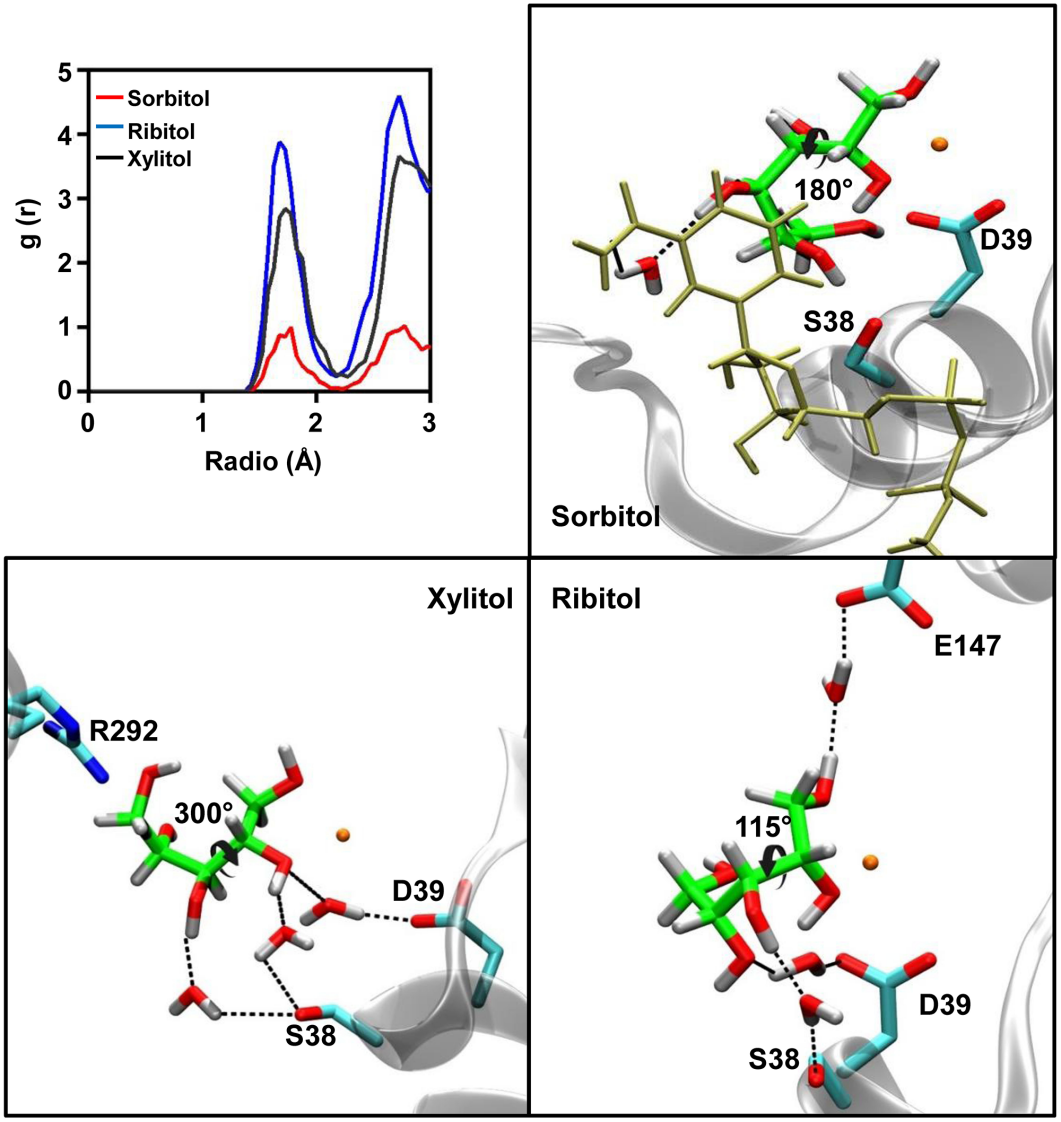
**Water molecules present in the binding pocket of sorbitol, ribitol, and xylitol.** The radial pair distribution function was calculated for the water molecules at different distances from the polyols in the active site of AtSDH (upper left panel). The remaining panels show the localization of the visually-tracked structural water molecules which mediate the interaction between the residues of AtSDH and the respective polyols. The colors of the atoms are the same as those described in **Figure 3**.

As shown in **Figure 5**, sorbitol and ribitol interact with residues of helix α1, whilst only xylitol forms its interactions with residues from the loop connecting helix α10 to strand β13. These divergent orientations originate from the different torsions adopted by the C2–C3 bond of the polyol structure. Upon measuring the dihedral angle along the C1–C2–C3–C4 bonds of the polyols (**Figure 6**), a value close to 180^∘^ is observed in the case of sorbitol, whilst in the case of ribitol it varies between 45^∘^ and 180^∘^ (with an average of 115^∘^) and it maintains around 300^∘^ in the case of xylitol.

We also observed the number of interactions that are mediated by water molecules. The radial pair distribution function was calculated for the probability of encountering a stable water molecule at different radii from the substrate along the complete trajectory (**Figure 6**). In the case of sorbitol, this analysis identified one water molecule mediating an interaction between the hydroxyl 4 and NAD^+^ carboxyamide. As such an interaction is not achieved with a protein residue, it strongly restrains the orientation of hydroxyls 5 and 6, for which direct stable interactions are formed with AtSDH. For xylitol and ribitol, the distribution functions indicate the establishment of almost 3 and 4 water-mediated interactions, respectively. Residues Asp39 and Ser38 in helix α1 seem to be important for the positioning of these structural waters. In the case of ribitol, Glu147, and hydroxyl 1 are bridged by structural water.

It is interesting that ribitol presents the lowest number of hydrogen bonds and the greatest quantity of structural water molecules, correlating well with the poorer kinetic performance of this substrate. On the other hand, sorbitol maintains the more stable interaction with the protein, and no water molecules interact via hydrogen bonds between the substrate and the enzyme. However xylitol, in addition to maintaining a number of hydrogen bonds not significantly different from the number observed for sorbitol, is also seen interacting through a high number of water-mediated interactions with residues present in helix α1 of AtSDH. When considering the differences between their specificity constants, it seems that xylitol is the preferred substrate of the enzyme given its higher number of direct and water-mediated hydrogen bond interactions. Sorbitol performs better in *k*cat than ribitol probably due to a greater stability in positioning the C2 carbon for hydride transfer to the nicotinamide moiety. These simulation results are also consistent with the preference of AtSDH for these substrate polyols in regard to the chirality of C2 and C4 (S and R, respectively). The opposite configuration in C2 would reorient the torsion angle along C1–C2–C3–C4 in order to maintain the bidentated zinc coordination, resulting in a complete loss of the observed interactions. The opposite configuration in C4 would disturb the pattern of hydrogen bonds with hydroxyls 4 and 5 in both xylitol and ribitol, and the ablation of the water-mediated interaction of sorbitol and NAD^+^ nicotinamide. Indeed, experimental findings demonstrate that L-arabitol [C-2 (S), C-4 (S)] and D-mannitol [C-2 (R), C-4 (R)] are oxidized at 59 and 32%, respectively, compared to sorbitol, by recombinant His-AtSDH (Aguayo et al., 2013).

Given these findings, whilst these polyols have been detected in different organs of *Arabidopsis* (Fiehn et al., 2000; Kaplan et al., 2004; Rizhsky et al., 2004; Bais et al., 2010; Ebert et al., 2010), it would be of particular interest to determine the effective intracellular concentrations of sorbitol, ribitol, and xylitol in plants grown under standard, and drought stress conditions, in order to discern which substrates are oxidized by AtSDH *in planta*.

## Conflict of Interest Statement

The authors declare that the research was conducted in the absence of any commercial or financial relationships that could be construed as a potential conflict of interest.

## Footnotes

1 http://www.genome.jp/kegg/pathway.html

2 http://www.rcsb.org

3 https://prosa.services.came.sbg.ac.at/prosa_help.html

## ABBREVIATIONS

AtSDH: *A. thaliana* sorbitol dehydrogenase
BaSDH: *B. argentifolii* sorbitol dehydrogenase
EDTA: ethylenediaminetetraacetic acid
HsSDH: *H. sapiens* sorbitol dehydrogenase
NAD^+^: nicotinamide adenine dinucleotide
PDB: Protein Data Base
T-CEP: tris(2-carboxyethyl)phosphine
TEV: tobacco etch virus.
