# Supplementary material for: Polyol specificity of recombinant Arabidopsis thaliana sorbitol dehydrogenase studied by enzyme kinetics and in silico modeling
Source: Front Plant Sci. 2015 Feb 23;6:91. doi: 10.3389/fpls.2015.00091 (PMC4337239; doi:10.3389/fpls.2015.00091)
Supplement: Supplementary file 1 [file table_1.docx]

**Supplementary Table 1. List of the closest AtSDH homologous structures found in PDB.**

BLAST parameters and ligand sites for the first six hits. The query sequence has a length of 364 residues.

| PDB ID | E-value | Coverage  (aa)^a^ | Identities  aa (%) | Ligand sites^b^ | | | | Structure name | Organism |
| --- | --- | --- | --- | --- | --- | --- | --- | --- | --- |
|  |  |  |  | Substrate | NAD | Zinc 1 | Zinc 2 |  |  |
| **1E3J** | **1.54E-93** | **348** | **177 (51%)** |  |  | **X** | **X** | **Ketose reductase (SDH)** | ***Bemisia argentifolii*** |
| 3QE3 | 3.19E-87 | 353 | 165 (47%) | ACY GOL |  | X |  | Sheep liver SDH | *Ovis aries* |
| 1PL7 | 1.27E-85 | 346 | 160 (46%) |  |  | X |  | SDH (apo) | *Homo sapiens* |
| **1PL6** | **1.27E-85** | **337** | **156 (46%)** | **572** | **X** | **X** |  | **SDH/NADH/inhibitor complex** | ***Homo sapiens*** |
| 1PL8 | 1.27E-85 | 346 | 160 (46%) |  | X | X |  | SDH/NAD^+^ complex | *Homo sapiens* |
| 3M6I | 1.78E-57 | 322 | 128 (39%) |  | X | X |  | L-arabinitol 4-dehydrogenase | *Neurospora crassa* |
| ^a^ amino acids. ^b^ ACY, GOL and 572 are PDB ligand identifiers for acetic acid, glycerol and 4-[2-(hydroxymethyl)pyrimidin-4-yl]-n,n-dimethylpiperazine-1-sulfonamide (CP-166,572), respectively. Zinc 1 denotes catalytic zinc. Zinc 2 denotes structural zinc. Bold hits indicate the structures used as templates for the modeling of AtSDH complexes. | | | | | | | | | |
